# Feature selection through validation and un-censoring of endovascular repair survival data for predicting the risk of re-intervention

**DOI:** 10.1186/s12911-017-0508-3

**Published:** 2017-08-03

**Authors:** Omneya Attallah, Alan Karthikesalingam, Peter J. E. Holt, Matthew M. Thompson, Rob Sayers, Matthew J. Bown, Eddie C. Choke, Xianghong Ma

**Affiliations:** 1 School of Engineering and Applied Science, Aston University, B4 7ET, Birmingham, UK; 2 Department of Electronics and Communications, College of Engineering and Technology, Arab Academy for Science and Technology, Alexandria, Egypt; 3 St George’s Vascular Institute, London, SW17 0QT UK; 4 St George’s Vascular Institute, St George’s University Hospitals NHS Foundation Trust, Blackshaw Road, London, SW17 0QT UK; 5 Vascular Surgery Group, University of Leicester, Leicester, UK; 6 Vascular Surgery Group, Robert Kilpatrick Clinical Sciences Building, Leicester Royal Infirmary, University of Leicester, Leicester, LE2 7LX UK

**Keywords:** Survival analysis, Censoring, Feature selection, Model selection, Factor analysis, Cox’s hazard proportional model, Endovascular aortic aneurysm repair

## Abstract

**Background:**

Feature selection (FS) process is essential in the medical area as it reduces the effort and time needed for physicians to measure unnecessary features. Choosing useful variables is a difficult task with the presence of censoring which is the unique characteristic in survival analysis. Most survival FS methods depend on Cox’s proportional hazard model; however, machine learning techniques (MLT) are preferred but not commonly used due to censoring. Techniques that have been proposed to adopt MLT to perform FS with survival data cannot be used with the high level of censoring. The researcher’s previous publications proposed a technique to deal with the high level of censoring. It also used existing FS techniques to reduce dataset dimension. However, in this paper a new FS technique was proposed and combined with feature transformation and the proposed uncensoring approaches to select a reduced set of features and produce a stable predictive model.

**Methods:**

In this paper, a FS technique based on artificial neural network (ANN) MLT is proposed to deal with highly censored Endovascular Aortic Repair (EVAR). Survival data EVAR datasets were collected during 2004 to 2010 from two vascular centers in order to produce a final stable model. They contain almost 91% of censored patients. The proposed approach used a wrapper FS method with ANN to select a reduced subset of features that predict the risk of EVAR re-intervention after 5 years to patients from two different centers located in the United Kingdom, to allow it to be potentially applied to cross-centers predictions. The proposed model is compared with the two popular FS techniques; Akaike and Bayesian information criteria (AIC, BIC) that are used with Cox’s model.

**Results:**

The final model outperforms other methods in distinguishing the high and low risk groups; as they both have concordance index and estimated AUC better than the Cox’s model based on AIC, BIC, Lasso, and SCAD approaches. These models have *p*-values lower than 0.05, meaning that patients with different risk groups can be separated significantly and those who would need re-intervention can be correctly predicted.

**Conclusion:**

The proposed approach will save time and effort made by physicians to collect unnecessary variables. The final reduced model was able to predict the long-term risk of aortic complications after EVAR. This predictive model can help clinicians decide patients’ future observation plan.

**Electronic supplementary material:**

The online version of this article (doi:10.1186/s12911-017-0508-3) contains supplementary material, which is available to authorized users.

## Background

Endovascular aortic aneurysm repair (EVAR) is a surgical operation for patients suffering from aorta inflation known as aorta aneurysm. EVAR carries significantly lower preoperative risk than open repair surgery; therefore, it is preferred by patients and recommended by medical guidelines as the choice for treating abdominal aortic aneurysm AAA [1]. There is an obligatory need for lifelong surveillance after this operation and it is considered to be expensive, varied, and poorly-calibrated [2]. However, the surveillance procedures are extensively various [3] and there is shortage of an indication to select the best timing or modality; Patients may be exposed to radiations and contrast nephropathy as a result of frequent surveillance. However, for some patients, complications required for treatment might be missed between surveillance [4–6]. For that reason, optimizing surveillance is very important [7, 8]. It is considered as an important issue in clinics and affects the long-standing cost-effectiveness of EVAR. By specifying which patients are more (high risk patients) or less likely (low risk patients) to require re-intervention within 5 years, a cost-effective and risk-stratified surveillance system could be achieved.

This study focuses on developing and validating a reduced predictive model for aortic complications after EVAR. In previous literature, models were usually built using only one dataset and validated with cross validation method [9–12]. However, cross-center testing is essential when the target is to validate the model for wider applications. Therefore in this work, two EVAR datasets were collected in two vascular centers in the United Kingdom which contain almost 91% of censored patients and which make the survival model construction and classification of difficult tasks. Censoring occurs when some patients could not be observed in the whole period of the survival study due to various reasons such as death, feeling better, or changing their residence location, leading to a type of missing data called censored data. The only information available for censored patients is the time until the last follow up or death (if it is not the event of interest). Therefore, the time until the event of interest is unknown [13]. The task here is to construct a predictive model using Center 1 EVAR data and testing it using other data collected from Center 2 with the presence of this high level of censoring. To deal with censoring, survival analysis techniques are adopted. It enables the use of the information available in the dataset even if it is censored. Actually, it does not omit censored patients [13]. Survival analysis techniques [14–16] were proposed with comparable datasets sizes to handle censoring.

Feature selection (FS) is very useful, especially in the medical area, as it reduces the time needed and the effort made by physicians to measure irrelevant and redundant features. It could avoid over-fitting that might occur during the learning process of the predictive model. It may also lower the model’s complexity and speed up the prediction process [17]. There are four primary approaches including filter, wrapper, embedded, and hybrid methods in FS [18]. These methods are used widely for standard data, though the task becomes more complex for survival data due the presence of censoring [17]. Therefore, the contribution of this paper is to select suitable feature selection methods for censored data, especially of a high level, which exist in the EVAR datasets. In FS using censored survival data, the most common approach used for modeling censored data is Cox’s proportional hazard model. Cox’s method uses the features information that might affect the hazard to build a predictive model [19]. Popular methods that perform FS using Cox’s include; wrapper FS methods which wrapped FS around Cox’s model and used several criteria such as Akaike or Bayesian information criteria, hazard ratio, and concordance index calculated from its prediction [20–23]. Some used Cox’s model as a filter approach and perform a univariate analysis to calculate Cox’s score metric for selection [24–28], while others used Wald test or likelihood test criteria instead [29] to quantify variables association with survival prediction. Several other metrics such as chi squared test [30], mutual information [31], and correlation information [32, 33] were also used to rank or filter variables. Other used penalized methods based on Cox’s model such as; penalized L1 Least absolute shrinkage and selection operator (LASSO) [34, 35–38]. It was then extended to adaptive LASSO, weighted LASSO, and gradient LASSO [39, 40–42]. However, Lasso was used with another survival model called accelerated failure time model instead of Cox [43] for constructing a survival model. Other penalized methods which perform feature selection include; elastic-net [44, 45] and smoothly absolute clipped deviation (SCAD) [46] models.

Machine learning techniques [20] (MLT), such as Bayesian networks and Artificial Neural Networks (ANN), are usually favorable over the standard statistical models such as Cox due to their ability to identify complex relations between data that improve prediction [47]. However, they cannot be used directly on survival data due to the presence of censoring. Therefore, several scenarios have been proposed to handle censoring, but these methods were not applied to perform FS [48].

Some papers have discussed the use of MLT to perform FS in survival analysis data with the presence of censoring. Among them are the popular partial logistic artificial neural network (PLANN) and its extension PLANN with automatic relevance detection (PLANN-ARD). PLANN was used with backward feature elimination [49]. PLANN-ARD [11] method selects features based on their relevance to the model according to a Bayesian framework. These methods handle censoring by dividing observation time into *n* intervals and repeating patients to these intervals. Repeating leads to unbalanced and biased predictive models, especially with high censoring level [50]. It also increases the complexity and training time of these models and may increase the noise level existing in the datasets. Authors in [28] applied Cox’s model to perform FS on censored data before entering a support vector machine (SVM) classifier. Others used wrapper FS methods [51, 52] with Bayes classifiers and K-nearest classifier. While in [30], a chi-square test was used to measure the degree of association between variables and observation time, then use ANN for prediction. Random survival forest classifier [53] is an extension to standard random forest classifier in which the output is survival time with a censor. It was used in [16, 54, 55] to perform FS. Gradient boosting with component-wise least squares was proposed by [56] based on cubic smoothing splines for L2 loss functions. It was then extended in [57] which illustrated that boosting technique works well in high-dimensional datasets for censored outcome data. Gradient boosting was used in [58] for survival analysis, however it differs from [57] in the choice of loss function, which in both cases is optimized via gradient boosting. The limitation of methods [11, 28, 30, 49–52] is the way they handled censoring which is carried out by deleting, ignoring, using only uncensored patients, or considering censored patients as event free. These methods of handling censoring are not appropriate to deal with a high level of censoring which exists in the suggested EVAR datasets. Moreover, the main drawback of tree based methods are instability, variable selection bias and over-fitting [59].

In the researcher’s previously published paper [60], an uncensoring approach was proposed to deal with the high level of censoring in the EVAR datasets using MLT without performing FS. Moreover, in the researcher’s other publication [61], the proposed uncensoring approach was combined with an existing ranking FS method to reduce the number of features in the datasets. Factor analysis is a feature transformation method used to transform data into a new domain so that most of the classification related information is compressed in a smaller number of features [62]. In this paper, it was used to group variables and to remove variables that are not related to any group, rather than to transform data into a new compressed domain used for classification. Moreover, a new FS technique was proposed to be combined with factor analysis and the researcher’s uncensoring approach to select a reduced set of features and produce a stable predictive model. A stable model means that there may be a slight or no change in the prediction error when the data examples used for its training are replaced by other cases [63]. Prediction error is decomposed into two types; errors due to variance and or to bias. In order to produce a stable algorithm, a tradeoff between variance and bias must be made [64]. Therefore, the new proposed FS approach addresses the instability issue to reduce bias and instability, which may be produced during FS and improves consistency of the feature selection using iterated nested cross validation method.

The proposed approach is divided into seven steps. The first step is feature reduction using factor analysis technique. The second is cross validation and permutation. The third is a stepwise feature selection which uses the *p*-value of the log rank test as a criterion. The fourth is the uncensoring step. The fifth is the iterated nested cross validation step followed by the sixth ANN model construction step. Steps two to six are repeated for each fold produced from the cross validation and permutation. They are repeated until a model with the minimum number of features is produced. The last step is the final model selection step done to choose among the different models constructed using each training fold generated in the cross validation step. The final model is the one which minimizes the *p*-value of the log rank test of the remaining censored test fold that was not used in training.

## Methods

### Data acquisition

From 2004 till 2010, follow up observations were collected to patients experiencing EVAR surgery from two different vascular centers located in the UK, at St George hospital (Center 1) and Leicester (Center 2). Both datasets consist of details of operative procedure and 47 patient morphological features. Three-dimensional computed tomography (CT) was employed to measure the pre-operational morphology features. The slice thickness of the CT images was 0.625 or 1.25 mm. They were obtained from the thoracic inlet to the level of the common femoral artery bifurcation. The total numbers of patients after removing missing values are 457 and 286 respectively, and the number of patients that experienced the EVAR re-intervention during the study period is 40 and 26 respectively for Centers 1 and 2. Details of these datasets can be found in a previous publication [65].

### Kaplan Meier curves

**Fig. 1:**
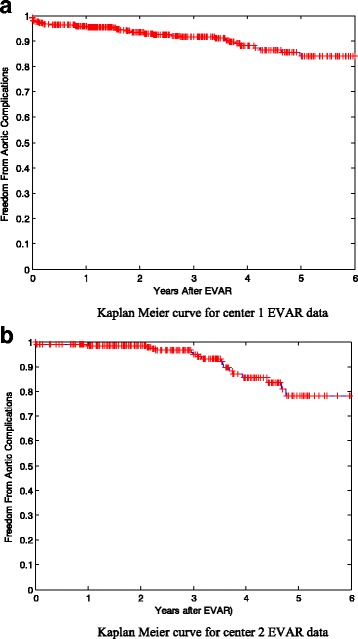
Kaplan Meier curve for **a** center 1 EVAR data **b** center 2 EVAR data

It is also known as product limit estimate of the survival function. It is one of the most well-known non-parametric survival analysis techniques. Non-parametric means that it does not take into account the information available by the predictive variables of a dataset when estimating survivability and does not assume survival distribution [66]. It calculates the probability of patient’s survival at any time for the whole dataset even if it is censored. Clinical trials adopt survival analysis techniques for several purposes, such as predicting survivability after treatment of a disease, recurrent of cancer, or the risk of re-intervention after a surgical intervention [67]. Kaplan Meier curves of Centers 1 and 2 are plotted before using any feature selection method. They are shown in Fig. 1a and b.

### Feature transformation and reduction

#### Factor analysis vs. principal component analysis

Principal Component Analysis (PCA) and Factor Analysis (FA) are the most well-known feature transformation and reduction techniques. PCA produces new features called components, which are the linear combinations of the original ones. These components are orthogonal to each other. They represent the variance in the data. Usually the first few components are the most important. However, FA seeks to determine the underlying structure among variables, which cannot be directly observed. It assumes that the attributes of data are produced by a linear combination of unobserved ones called latent factors. There are two types of factors, common and unique. The common factor is an unobserved variable that expresses the common variance between two or more observed variables (original measured variables). The unique variance is an unobserved variable that explains the variance of only one observed variable. Features that are not correlated to any factor may be considered as unimportant and may be removed from the data. Therefore, FA is favorable than PCA as it aims to find the minimum number of factors that explain the underlying structure of the data [68]. Additionally, PCA does not take into account the effect of unique variance in determining principal components; it only regards the common variance. FA considers both the common and unique variances in calculating latent factors.

#### Factor rotation

FA seeks to reduce the dimension of data in order to visualize and understand grouping and underlying structures between variables. Though, sometimes the primary latent factors are not able to clearly demonstrate this. Therefore, factor rotation may be needed to solve the problem. Rotation process is applied to the primary factors by rotating them into new axes called rotated factors [69]. These new axes produce large loading factors on one or two of the rotated factors, and small loading factors on others. The varimax and promax rotations are the most popular rotation techniques. A varimax rotation creates orthogonal uncorrelated rotated factors, while promax produces oblique ones [70]. In some cases, a promax rotation is favorable over varimax as it is a faster and simpler method [71]. However, in this paper varimax rotation was used to generate uncorrelated rotated latent factor in order that the variable related to different latent factors will be uncorrelated.

### Cox proportional hazard model

Cox-proportional hazard or Cox regression is the most popular semi-parametric method used in clinical trials. It builds a hazard model using covariates values by assuming that their effect on survival function is constant over time [72]. The relationship between patients’ survival and covariates are investigated using Cox’s model. In other words, it is used to estimate the risk of event given the prognostic variables at a time *t*. The model’s output will be a hazard as a function of time and specific covariates.

### Bayesian networks

Bayesian network follows a particular structure of probabilistic graphical models known as directed acyclic graph (DAG). A DAG consists of a number of nodes (vertices) *V* = {*V*_1_, *V*_2_, *V*_3_, . … *V*_*n*_} representing variables and arcs *A* ⊆ *V* × *V*connecting them and representing conditional and unconditional dependencies between these variables. Each node *V* represents a specific random variable and is drawn as a circle with its name on it. Arcs connecting the nodes are drawn as arrows and must be directed in only one direction which means that when an arc leaves a node, it does not return to it again [73]. Each node *V*_*i*_ can be considered as a parent node when an arrow comes out of it. It can also be a child node when an arrow points towards it. BN illustrates relations and joint probabilities among variables of a dataset. This interpretability and its ability to provide reasoning with uncertainty make it appropriate to be used in the proposed approach.

### Artificial neural network

Artificial neural networks (ANNs) is one of the most popular and widely used ML techniques, especially in the medical area, as it has high ability to get good prediction even with noisy data [74] like the endovascular aortic repair (EVAR) datasets used in this paper. The artificial neural networks (ANN) are models stimulated by the biological nervous system of animals and specifically the neural networks of the brain [75]. Multilayer perception MLP-ANN is the commonly used ANN in the medical area. It consists of a number of neurons referred as nodes connected together by weights. These nodes are gathered together to form N layers consisting of one input, one output, and one or more hidden layers. The learning process in ANN is done by changing the weights of the connected neurons with the help of the training data and a learning algorithm.

### The proposed algorithm

The proposed technique used Center 1 (457 patients) for feature selection and Center 2 (286 patients) for its validation and assessment.

**Fig. 2:**
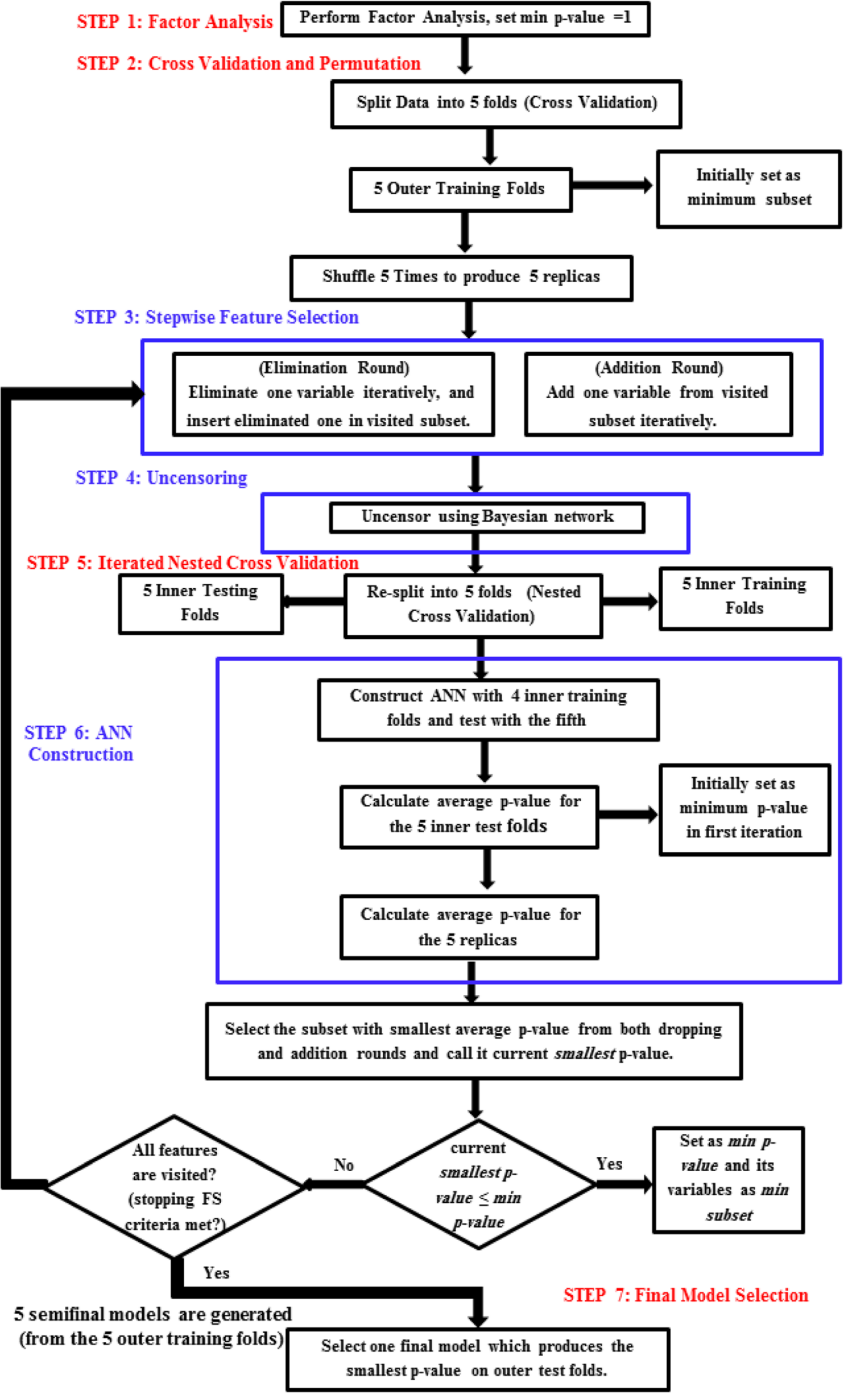
Flow chart of the proposed algorithm

The proposed algorithm is divided into seven main steps. The flowchart in Fig. 2 describes the subsequent procedures. It also illustrates the main three areas of contributions of the proposed approach which are feature selection, uncensoring, and classification with their interactions which are highlighted in blue. Step 1 is reducing features using factor analysis. It applies factor analysis technique in order to lower the dimension of the data as an initial step. This was done after using Kaiser-Meyer-Olkin and Bartlett’s tests to examine whether FA is needed for the data [76, 77]. Step 2 is the cross validation which splits Center 1 data into five outer training and testing folds and this is called the outer loop. It is used in the feature selection process for choosing the final reduced model, which produces the smallest *p*-value of the log rank test. Step 3 is the feature selection process which uses the *p*-value of the log rank test to select the variables used to build the ANN model from the uncensored training folds of Center 1 data. Step 4 is the uncensoring of the EVAR data. Step 5 is the iterated nested cross validation; which re-splits each outer training fold into five inner (nested) training and testing partitions. This is called the inner loop and it was used to overcome overoptimistic prediction that may occur during feature selection, uncensoring and ANN construction steps. Moreover, this process produces a stable algorithm that tunes the tradeoff between variance and bias [78, 79]. Step 6 is the ANN model construction used for predicting the risk of re-intervention. Step 7 is to select the final model with the optimal number of features; the chosen model is the one which minimizes the *p*-value of the log rank test on the remaining testing fold which has not been used in the FS and ANN construction process. This *p*-value is used to determine whether the ANN model built with the selected features was capable of differentiating between the two risk groups of the censored Center 2 EVAR data. Usually, when it is lower than a significance level of 0.05, the two risk groups are considerably different, discriminative and separable. In the following subsection, each step will be discussed in detail.

#### Feature reduction using factor analysis step

**Fig. 3:**
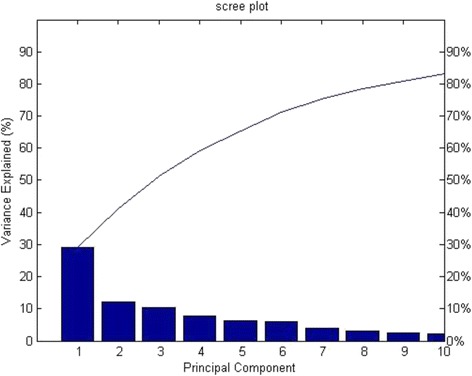
Scree Plot of the PCA using Center1 EVAR data

FA was employed in this work as a first step to reduce the dimension of the datasets by eliminating the variables that are not related to any latent factor. This will reduce the computational cost required later in the proposed feature selection algorithm. The number of latent factors has to be determined first in order to start FA. In order to determine the number of latent factors used in factor analysis, a scree plot is produced which shows the Eigen values accompanied with principle components or latent factor listed in descending order versus the number of components or factors as shown in Fig. 3, only 6 components showed almost 80% of the variance in the data. Therefore, the number 6 is adopted to do FA on Center 1 data.

**Fig. 4:**
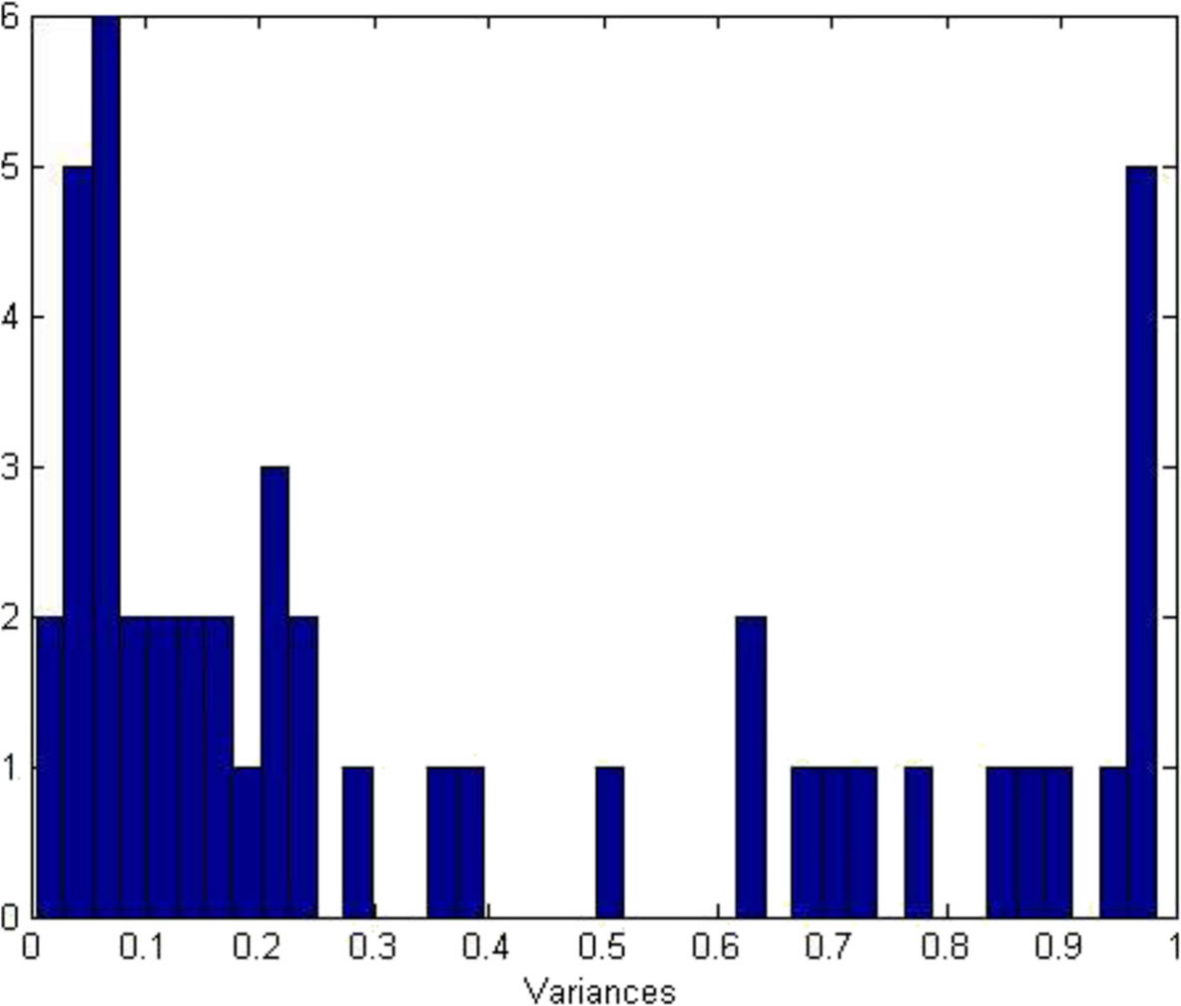
Histogram of the Uniqueness values for center1 EVAR at attributes

After applying FA on the data, insignificant variables should be removed from the dataset. The most common criterion used to drop these variables is communality which is the part of the variance generated from the common factors. It is calculated by summing the squared factor loadings of all common factors for a given variable [80]. Factor loadings are the values or weights (called loadings) multiplied by each factor, then added together to form a linear combination of factors that produce observed variables. As a rule of thumb, when a variable has large loadings to some factors, this means that this variable can be represented by particular factors. Therefore, it should be retained as it is considered an influential observed variable [81]. For this reason, communality of high values is better. If the communality of a variable is high, this means that the factors describe a large percentage of the variable’s variance. Hence, this indicates that this specific variable is well explained by the common factors, and therefore that the factor analysis is reliable. Low communality of a variable indicates that this variable does not load to any factor (have low loading factors), can be removed, and is considered a non-influential variable [81, 82]. Communality equals to (1- uniqueness). The uniqueness is a metric which measures the portion of the variance produced due to unique factors. In this work, the threshold of communality according to which features are dropped is determined from the histogram of unique variances (uniqueness), produced by unique factors. Variables with uniqueness values greater than this threshold will be removed, as they correspond to low communalities. As shown in Fig. 4, the first sudden drop in the uniqueness occurs at 0.25. The researcher has chosen this threshold as other greater values correspond to high uniquenesses and low communalities.

#### Cross validation and permutation step

Center 1 EVAR data is divided into five folds using five folds cross validation method. Each separate four folds are called the outer training fold and the fifth is called the outer testing fold. Each outer training fold is used in nested cross validation, stepwise feature selection, uncensoring, and ANN construction steps later. During the stepwise feature selection step, it is permuted five times to produce its replicas after being uncensored (in the uncensoring step).

#### Stepwise feature selection step

In the stepwise feature selection (SWFS) step, a canonical greedy stepwise search was used to select features using the *p*-value of the log-rank test as a performance metric. It is divided into two stages; feature elimination and addition. The main advantage of this strategy is that the eliminated features are given another chance to re-enter the feature selection process. It starts with the elimination stage in which all the features of each outer training fold is eliminated iteratively and the rest are used in the uncensoring and ANN construction steps after being permuted and re-split into inner nested folds to calculate the average of the *p*-value of the log-rank test. This prevents over-fitting and overoptimistic *p*-value predictions and enables the production of a stable model. The subset with the smallest averaged *p*-value is the one chosen and called “minimum subset” and its average *p*-value is called the “minimum averaged *p*-value”. Features eliminated will be inserted in a subset called the “visited subset” and will be given another chance to enter the FS process again in the addition stage. This is done by adding features of the visited subset iteratively, and then repeating the uncensoring and ANN construction steps. If the subset with the smallest average *p*-value has a *p*-value lower than the “minimum averaged *p*-value”, then this subset is set as the “minimum subset” and its *p*-value as the new “minimum averaged *p*-value”. This procedure is repeated until all features are visited.

#### Uncensoring step

The censoring time variable for each outer training loop was used to divide patients into three groups. The first one belongs to patients that experience the re-intervention at a time shorter than or equal to 5 years (re-intervened or high risk patients). The second group refers to patients that did not need the re-intervention for longer than 5 years (low chance of re-intervention or low risk patients). Finally, the rest of the patients are considered as the censored group which is the third group (those who died or left the follow up observations before 5 years).

Afterwards, each outer training fold was discretized using equal width unsupervised discretization technique. Then, each risk group was employed to build two Bayesian networks called low and high risk networks *B* ^*low*^ and *B* ^*high*^_,_ respectively. Each Bayesian network is a directed acyclic graph (DAG) given a symbol *ξ*. They are constructed using the input variables *V*. Each variable *V*_*i*_ represents a node in this network and its parent is defined by *π*. The Bayesian networks were learned with Hill climbing structure learning algorithm [83]. The scoring function used for choosing the structure of the network has a minimum description [84]. Parameter learning was done using a maximum likelihood procedure to determine relation between nodes of a network [85].

The output class of each risk group is then removed as it is already known in the network. Next, each censored patient from the censored group is compared with the intrinsic distribution *p* of each network *p* ^*high*^ and *p* ^*low*^_,_ correspondingly. Likelihood *ℓ*(*x*_*c*_/*p*) of the censored patients belongs to which network is used to uncensor the patient and determine to which group he/she belongs. It is calculated using Eqs. (1) and (2)

1 $$ \widehat{\ell}\left({x}_c/{p}^{high}\right)=\ell \left({x}_c/{B}^{high}\right)=p\left({x}_c/{\xi}^{high},{p}^{high}\right)=\prod_{i=1}^n{p}^{high}\left({V}_i/\pi \left({V}_i\right)\right). $$

2 $$ \widehat{\ell}\left({x}_c/{p}^{low}\right)=\ell \left({x}_c/{B}^{low}\right)=p\left({x}_c/{\xi}^{low},{p}^{low}\right)=\prod_{i=1}^n{p}^{low}\left({V}_i/\pi \left({V}_i\right)\right). $$

Afterwards, the posterior probability that outcome predictions that patients belong to each network given that they are censored (*x*_*c*_) *P*(*O*/*x*_*c*_) in Eq. (5) is calculated using Eqs. (3) and (4).

3 $$ P\left({O}^{high}/{x}_c\right)=\widehat{P}\left({O}^{high}\right)\ast \frac{\widehat{\ell}\left({x}_c/{p}^{high}\right)}{P\left({x}_c\right)}. $$

4 $$ P\left({O}^{low}/{x}_c\right)=\widehat{P}\left({O}^{low}\right)\ast \frac{\widehat{\ell}\left({x}_c/{p}^{low}\right)}{P\left({x}_c\right)}. $$

5 $$ P\left(O/{x}_c\right)=P\left({O}^{high}/{x}_c\right)+P\left({O}^{high}/{x}_c\right)=\frac{\widehat{P}\left({O}^{high}\right)\ast \widehat{\ell}\left({x}_c/{p}^{high}\right)+\widehat{P}\left({O}^{low}\right)\ast \widehat{\ell}\left({x}_c/{p}^{low}\right)}{P\left({x}_c\right)}, $$

Equation (5) is then normalized to ignore the effect of probability of a censored instance *P*(*x*_*c*_) by dividing Eq. (5) by *P*(*O*/*x*_*c*_)*P(*x*_*c*_) to get Eq. (6).

6 $$ P\left({O}^{high}/{x}_c\right)+P\left({O}^{low}/{x}_c\right)=1. $$

Finally, a threshold named censoring correction *P*_*Th*_ is selected to determine whether each censored patient belongs to the high or low risk groups. If *P*(*O* ^*high*^/*x*_*c*_) is bigger than *P*_*Th*_, the censored patient will be considered as high risk and vice versa.

#### Iterated nested cross validation step

After uncensoring all the replicas of each outer training partition, each replica is divided into five nested folds. Every different four folds are called inner training folds and are used to build an ANN (in the ANN construction step), while the fifth one is called inner test fold and is used for measuring the performance of the ANN using the *p*-value of the log-rank test of this inner test set. This is done for the different inner training and testing folds. Afterwards, the average of the *p*-value is then calculated. This process prevents overoptimistic results. It is worthy of note that each outer training fold was used to construct the ANN with different number of neurons. The process is repeated for every replica of the outer training fold, and then the average of all of them is calculated to produce the *p*-value of the nested cross validation which is used in the stepwise feature selection step as a criterion to select the attributes. It produces a stable model that tunes the tradeoff between variance and bias. Nested cross validation is also used as a method of avoiding over-fitting used due to the small sample size available in the data.

#### ANN construction step

A three layer MLP-ANN trained with gradient descent with momentum back propagation algorithm was used to build a model for every outer training fold with different number of neurons. Sigmoid was used as an activation function. Other parameters are kept with their default values. Each model was used in the feature selection process to determine which one has the optimal features that minimize the *p*-value. The model has the ability to distinguish between the two risks groups of Center 1 data used in the ANN construction and can be validated with Center 2 data. The k-fold cross validation procedure was used to select the number of hidden neurons that reduces chances of over-fitting. In addition, it was employed and combined with feature selection and iterated nested cross validation to reduce chances of over-fitting.

#### Final model selection step

Finally, five models will be produced from the five outer training folds that were used to build an ANN with different numbers of neurons. Each one is tested with the outer test fold which is not used in the ANN construction and the feature selection steps. The model that produces the minimum *p*-value in the outer test fold is the one chosen as a final model. The number of features and neurons produced in this model is used to train an ANN using Center 1 data. Center 2 data is used to validate the selected model.

## Results

### Results of the proposed feature selection method

One common approach for feature selection is to utilize the whole data set for the selection procedure. Re-sampling techniques such as cross validation and bootstrapping can split data into separate parts. Parts will be used for feature selection and evaluation. The other part that is not used in model construction will be used for validation and assessing the performance of the final feature selected model. In the proposed algorithm, Center 1 data were used for the feature and model selection (number of hidden neurons of ANN), while Center 2 data were employed for the validation and assessment. Cross and nested cross validation re-sampling techniques were used to split Center 1 data. The inner nested loop was used for producing an unbiased, stable algorithm and overcoming over-fitting and the overoptimistic predictions that might be produced. The outer loop was used to choose the model that produces the smallest *p*-value of the log-rank test on the outer test set. It is worth mentioning that the ANN of final chosen model has the number of neurons equal to seven.

Table 1 shows the results of using all the features available in Center 1 data and the results after FA reduction and the stepwise FS steps. It is clear from Table 1 that the number of features has been reduced from 45 to 27 after the FA step. The CI for Center 2 prediction has increased from 0.6 to 0.61. The *p*-value for Center 2 has improved from 0.036 to 0.034. It is also obvious from the table that the number of features selected in the final model after the stepwise selection step is lowered to 7. The *p*-value and CI for Center 2 predictions have improved to 0.022 and 0.63, respectively.

**Table 1:** Results of the proposed algorithm with the real dataset

The proposed algorithm	Number of features	*p*-value (Logrank)	CI(standard error SE)	Sensitivity
All Features	45	0.036	0.6 (0.0677)	0.46
FA Features	27	0.034	0.61(0.0715)	0.57
Stepwise selection Features	7	0.022	0.63 (0.0739)	0.73

### Results of the final model of the proposed method compared to Cox’s models based on AIC, BIC, lasso, and SCAD

In this section, the results of the final model produced by the proposed algorithm are compared with the most well-known, semi-parametric method used in clinical trials, Cox’s Hazard proportional model. AIC, BIC, Lasso, and SCAD are popular feature and model selection techniques that are used with Cox’s model to produce a stable algorithm with reduced number of features. It is well known that the Cox’s output is continuous. In order to convert this output to binary outcome indicating the risk group, the parameter estimates from the final models were multiplied by each variable to generate a risk score and a threshold value is used to separate risk groups. A value above the threshold indicates high risk (class value of 1) and vice versa. The threshold that separated the two risk groups for Center 1 was 3.1 using AIC which is equivalent to the mean value of the risk score. The thresholds for BIC, Lasso, and SCAD are 2.4, 6.3, and 1.8, respectively. The same threshold is applied to Center 2 data. Tables 2 and 3 show the results of the proposed algorithm compared with the results of AIC, BIC, Lasso, and SCAD for Centers 1 and 2, respectively. The hyper parameter of Lasso and SCAD methods was optimized using ten-fold, cross validation.

**Table 2:** Results of center1 data using the proposed algorithm compared with Cox’s model using AIC, BIC, Lasso, and SCAD

Algorithm used	Number of features	*p*-value (Logrank)	CI(SE)	Sensitivity
Proposed Algorithm	7	<0.00001	0.74 (0.0439)	0.76
AIC Algorithm	14	<0.00001	0.79 (0.0408)	0.69
BIC Algorithm	14	<0.00001	0.76 (0.0465)	0.38
Lasso Algorithm	7	<0.00001	0.7382 (0.0426)	0.714
SCAD Algorithm	3	0.0078	0.6271 (0.0518)	0.643

**Table 3:** Results of center2 data using the proposed algorithm compared with Cox’s model using AIC, BIC, Lasso, and SCAD

Algorithm used	Number of features	*p*-value (Logrank)	CI(SE)	Uno’s AUC	Sensitivity
Proposed Algorithm	7	0.022	0.63 (0.0739)	0.612	0.73
AIC Algorithm	14	0.034	0.61 (0.0725)	0.60	0.35
BIC Algorithm	14	0.029	0.63 (0.0685)	0.605	0.23
Lasso Algorithm	7	0.0068	0.615 (0.0864)	0.627	0.5
SCAD Algorithm	3	0.2759	0.592 (0.0658)	0.58	0.6923

It is obvious from Table 2 that the proposed algorithm, AIC, BIC, Lasso, and SCAD Cox’s algorithm have all successfully distinguished the two risk groups of Center 1 as they all have *p*-value lower than 0.00001 except for SCAD which has a *p*-value of 0.0078. These *p*-values are beyond the significant level 0.05. The CI of AIC, BIC, Lasso, SCAD Cox’s models are 0.79, 0.76, 0.7382, and 0.6271, which are greater than the proposed algorithm 0.74 except for the SCAD (0.6271). However, the proposed algorithm outperforms the AIC and Cox algorithm in the number of features selected in the final model which is 7, while in the other two methods it is 14. The proposed algorithm has the same number of selected features as Lasso [7], but greater than SCAD [3]. The advantage of using the proposed algorithm over the other methods in predicting the risk of re-intervention appears clearly in the predictions of Center 2 as shown in Table 3. First, it has the same CI 0.63 as the BIC model and outperforms the AIC, Lasso, and SCAD with CI equals to 0.61, 0.615, and 0.592. Moreover, it has an AUC (0.612) which is better than that of AIC, BIC, and SCAD (0.6, 0.605, and 0.58, respectively). However, the AUC of the proposed method is lower than that of Lasso 0.627. Finally, the *p*-values of the proposed method, AIC, BIC, Lasso, and SCAD algorithms are 0.022, 0.034, 0.029, 0.0068, and 0.2759. These *p*-values indicate that the results of all models can be separated significantly to distinguish between the high and low risk groups except for SCAD which has *p*-value of 0.2759 which is greater than 0.05.

**Fig. 5:**
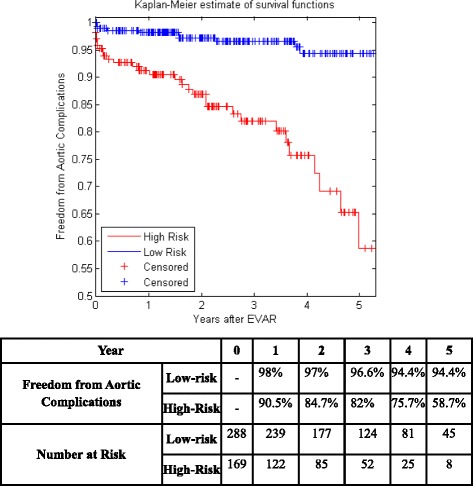
Kaplan Meier curves of the risk groups of center 1 prediction using the proposed algorithm

**Fig. 6:**
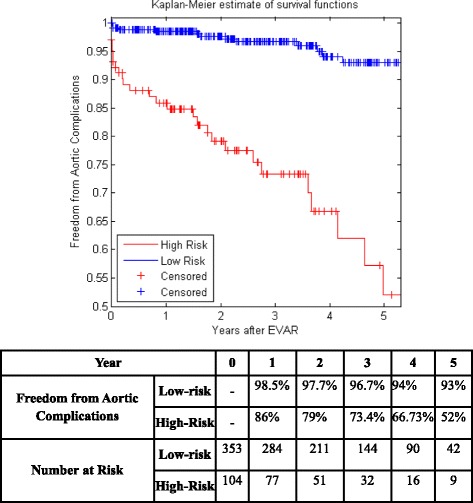
Kaplan Meier curves of the risk groups of center 1 predictions using the AIC –Cox’s model

**Fig. 7:**
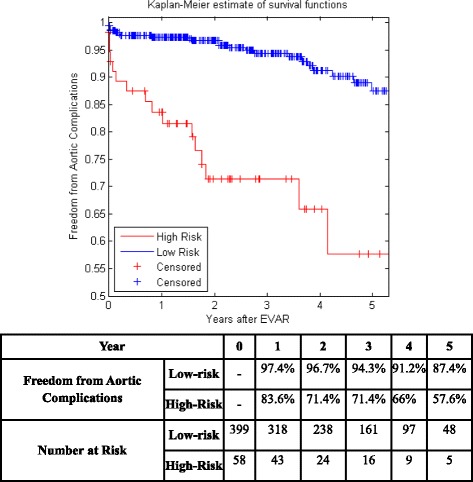
Kaplan Meier curves of the risk groups of center 1 predictions using the BIC –Cox’s model

**Fig. 8:**
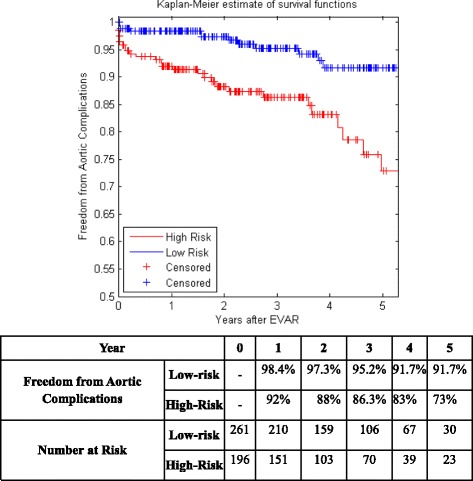
Kaplan Meier curves of the risk groups of center 1 predictions using the Lasso –Cox’s model

**Fig. 9:**
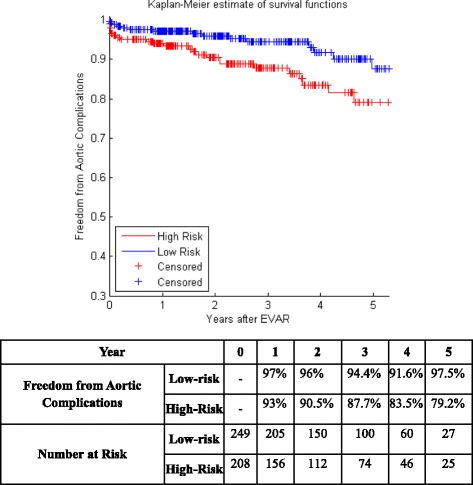
Kaplan Meier curves of the risk groups of center 1 predictions using the SCAD –Cox’s model

**Fig. 10:**
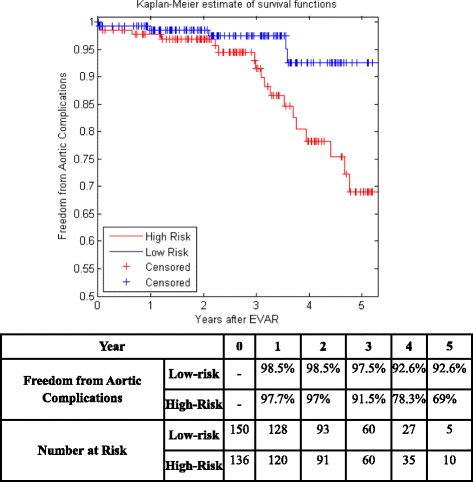
Kaplan Meier curves of the risk groups of center 2 predictions using the proposed algorithm

**Fig. 11:**
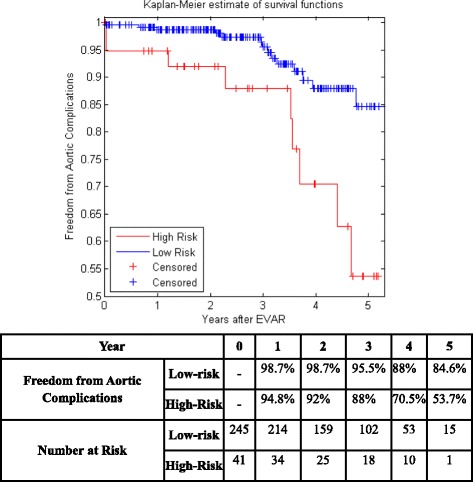
Kaplan Meier curves of the risk groups of center 2 predictions using the AIC –Cox’s model

**Fig. 12:**
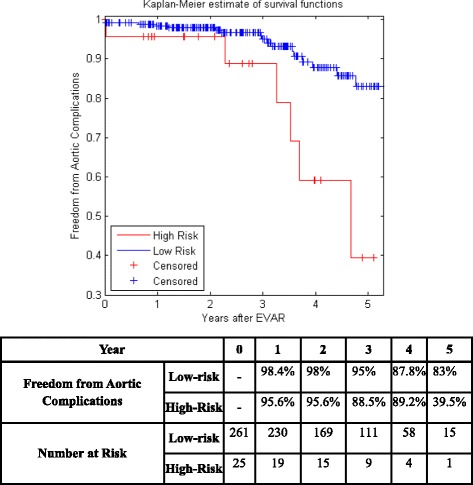
Kaplan Meier curves of the risk groups of center 2 predictions using the BIC –Cox’s model

**Fig. 13:**
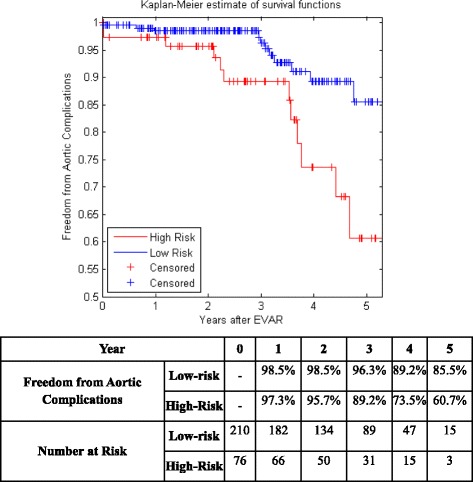
Kaplan Meier curves of the risk groups of center 2 predictions using the Lasso –Cox’s model

**Fig. 14:**
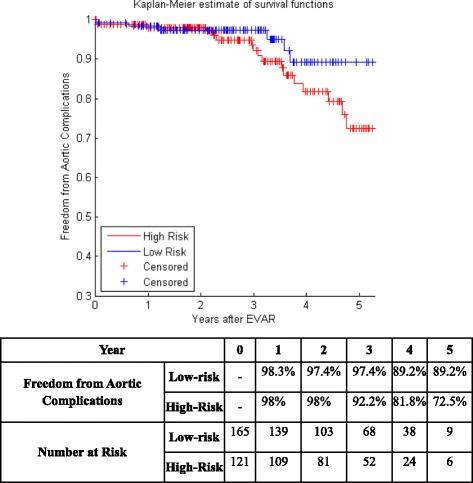
Kaplan Meier curves of the risk groups of center 2 predictions using the SCAD –Cox’s model

Figure 5 shows the KM curves of the two risk groups using the final selected model of the proposed algorithm for Center 1 compared with AIC (Fig. 6), BIC (Fig. 7), Lasso (Fig. 8), and SCAD (Fig. 9), respectively. Figure 10 shows the KM curves of the two risk groups using the final selected model of the proposed algorithm for Center 2 compared with AIC (Fig. 11), BIC (Fig. 12), Lasso (Fig. 13), and SCAD (Fig. 14), correspondingly. Moreover, these figures include the probability of freedom from aortic complications within the 5 years after EVAR along with the number of patients at risk of each group (low and high risks).

Figure 5 also shows that the proposed algorithm classified 169 of the Center 1 patients as high risk, which is equivalent to 37% of the patients. Freedom from aortic complications of the high risk patients reached 58.7% vs. 94.4% in low risk patients in the fifth year as shown in Fig. 5 (*p* < 0.00001 log-rank test). However, the AIC, BIC, Lasso, and SCAD Cox’s model predicted 104, 58, 196, and 208 patients as high risk as shown in Figs. 6, 7, 8 and 9. These are equivalent to 23, 13, 42, and 45.5% of the patients. Freedom from aortic complications of the high risk patients reached using AIC, BIC, and Lasso are 52% vs. 93%, 57.6% vs. 87.4%, and 91.7% vs. 73% in low risk patients in the fifth year (*p* < 0.00001 log-rank test). For, SCAD, the high risk is 79.2% vs. 97.5% of the low risk in the fifth year (*p* = 0.0078 log-rank test).

Figure 10 shows that the proposed algorithm classified 136 of the Center 2 patients as high risk, which is equivalent to 47.5% of the patients. Freedom from aortic complications of the high risk patients reached 69% vs. 92.6% in low risk patients in the fifth year (*p* < 0.022 log-rank test). However, the AIC, and BIC Cox’s model predicted 25 and 41 patients as high risk as shown in Figs. 11 and 12, which is equivalent to 8 and 14.3% of the patients. Freedom from aortic complications of the high risk patients reached 39.5% vs. 83% and 53.7% vs. 84.6% in low risk patients in the fifth year (*p* = 0.034 and 0.029 log-rank test). Lasso and SCAD algorithms predicted 76 and 121 patients as high risk as shown in Figs. 13 and 14, which is equivalent to 26.5 and 42.3% of the patients. Freedom from aortic complications of the high risk patients reached 60.7% vs. 87.5% for low risk in Lasso and 72.5% vs. 89.2% in low risk patients in SCAD in the fifth year (*p* = 0.034 and 0.029 log-rank test).

## Discussion

The influence of aortic morphology on long term prediction of EVAR is complicated and suitable to be analyzed with ANN, with significant possible interface between aortic volume, shape, diameter, and angulation. Current approaches have shown that aneurysm diameter predicts re-intervention after EVAR [86, 87], however evidence also recommended that other features of aneurysm morphology influence long-term clinical success [88–90]. Further complex concerns such as endograft configuration and deployment, or intermediate markers of patients’ cardiovascular risk phenotype, could possibly be used to train ANN in prospective studies, which increases the clinical significance of prediction and makes it more reliable. Also, adding more operative factors such as graft size, and endoleak at completion or post-operative variables, such as endoleak at early surveillance scans, could significantly enhance the discriminatory power of ANNs [61]. As an evidence, the proposed FS approach using ANN has selected maximum aneurysm neck diameter, diameter of the left common iliac artery 1 and 5 mm below internal iliac ostium, maximum common iliac artery diameter 5 mm proximal to internal iliac origin, maximum iliac tortuosity index, maximum common iliac thrombus volume, and right common iliac artery non luminal volume. These features were examined by clinicians who approved that they have outstanding validation terms for the prediction morphology for current endografts available. Results were compared with current clinical method such as; SGVI (St George Vascular Institute), which showed that the proposed method has superior performance. The seven features ANN of the proposed method have greater predictive ability in classifying low and high risk patients than the SGVI score [91]. Concordance index, estimated AUC, and *p*-value of the predictive model using the selected features show good clinical sense. In addition, they potentially indicate an increase in the event detection (EVAR re-intervention in this study) and risk group separation without affecting the cost of collecting unnecessary additional variables and surveillance cost.

Details about the uncensoring algorithm, could be found in [60]. Both Bayesian and neural network were constructed using Weka software [92]. Bayesian network is used in this paper as it helps physicians and clinicians to understand the relations between variables. It is a graphical probabilistic network and it determines the joint probabilities between the variables of a dataset. Each variable is given as a node in the network. The final graph makes it easier for doctors to figure out the relations between the variables and how predictions are performed, as they are less aware of data mining and machine learning techniques [31]. The structures of the Bayesian networks generated after feature selection can be found in Additional file 1.

This work has several contributions; the first one is to use machine learning techniques such as Bayesian networks and Artificial Neural networks to solve censoring issue and build a predictive model. As mentioned before, previous techniques that used machine learning techniques dealt with low to medium level censoring. Moreover, the way they handled censoring may lead to a biased predictive model, especially with high censoring. The second one is to deal with the high censoring issue which is a major difficulty in datasets that prevent building a predictive model capable of predicting the risk of EVAR re-intervention. Since most of the previous studies used only one dataset split using k-fold cross validation or bootstapping to validate the results. The third contribution is to justify the model in future cross-center applications by constructing a predictive model using Center 1 EVAR data and testing them using other data collected from Center 2. Earlier work was mainly concerned with dealing with the censoring issue without performing FS. Existing FS methods dealing with censored data have several drawbacks and cannot be used with the highly censored EVAR datasets as mentioned before in the introduction. The fourth one is to apply the appropriate feature selection techniques to the highly censored endovascular repair survival data.

The proposed algorithm used Bayesian network to uncensor the highly censored EVAR datasets and ANN for prediction of risks. It employed FA reduction and stepwise FS to reduce the number of features used for building the predictive model. It used cross validation for model selection (choosing the number of neurons of ANN) and iterated nested cross validation to generate a stable feature selection algorithm and produce the final reduced model. A simulation study was performed and included in the Additional file 2 to show the effectiveness of the proposed algorithm compared with other variable selection methods.

## Conclusion

A new feature selection technique was proposed to build and validate a predictive reduced model using the two EVAR datasets (743 patients) collected during 2004 to 2010 from two vascular centers. The final reduced model was able to predict the long-term risk of aortic complications after EVAR. Only morphology features were used for constructing the model as they have greater effect on aortic complications than physiology ones [6, 93, 94]. The proposed feature selection technique has successfully reduced the final model to 7 features only instead of the full model of 45 attributes. The final reduced model was validated using Center 2 data and the results showed that it was capable of predicting the risk of EVAR re-intervention. It was also able to successfully distinguish between the two risk groups of each center as the *p*-value of the log rank test was lower than 0.00001 for Center 1 and 0.022 for Center 2. This proves that the model can be used in cross-center predictions. This will help clinicians to put a future different follow up surveillance plan for different risk groups of EVAR patients.

Four other popular feature selection techniques AIC, BIC, Lasso, and SCAD were compared to the proposed algorithm. The reduced predictive model constructed using the proposed approach has higher ability in discriminating and distinguishing between risk groups of patients than other variable selection methods based on Cox’s model, since it has better concordance index, and estimated AUC. In addition, the number of patients of Center 1 that were classified as high risk using the proposed method, AIC, BIC, Lasso, and SCAD Cox’s models are 169, 104, 58, 196, and 208 patients versus 136, 25, 41, 76, and 121 for Center 2. This means that the proposed algorithm better identifies the risk of EVAR re-intervention. Therefore, it may be preferred by doctors to decide which surveillance plan each patient should undertake. Clinicians will put a more regular monitoring schedule in the future follow up and surveillance plan for those who have high risk of needing re-intervention, and the lower risk patients can be monitored less regularly. This would help in balancing and developing a cost-effectiveness surveillance system.

## Additional files

Additional file 1: The structures of the high and low bayesian networks after feature selection. (PDF 208 kb)

Additional file 2: Comparative variable selection survival models and the generated Simulation Study. (PDF 180 kb)

## Abbreviations

AAA: Abdominal aortic aneurysm
AIC: Akaike information criteria
ANN: Artificial neural network
BIC: Bayesian information criteria
BN: Bayesian network
BP: Back propagation
CI: Concordance index
CT: Computed tomography
DAG: Direct acyclic graph
EVAR: Endovascular aortic repair
FA: Factor analysis
FR: Feature ranking
FS: Feature selection
KM: Kaplan Meier
Lasso: Least absolute shrinkage and selection operator
MLP: Multilayer perceptions
MLT: Machine learning techniques
MPL: Maximum partial likelihood
PCA: Principal component analysis
PH: Proportional hazard
PLANN: Partial logistic regression artificial neural network
REINT: Risk of endovascular aortic repair intervention
SCAD: Smoothly clipped absolute deviation
SWFMS: Stepwise feature model selection
