# Supplementary material for: Feature selection through validation and un-censoring of endovascular repair survival data for predicting the risk of re-intervention
Source: BMC Med Inform Decis Mak. 2017 Aug 3;17:115. doi: 10.1186/s12911-017-0508-3 (PMC5543447; doi:10.1186/s12911-017-0508-3)
Supplement: Supplementary file 1 — The structures of the high and low bayesian networks after feature selection. (PDF 208 kb) [file 12911_2017_508_MOESM1_ESM.pdf]

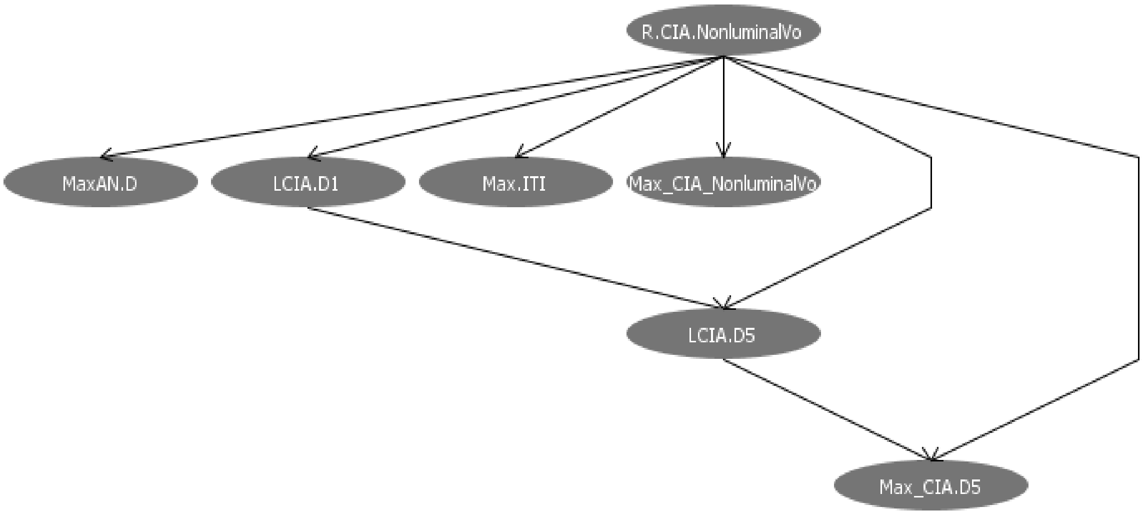


Figure S 1. The low risk Bayesian network constructed using patients of the low risk group of the final seven reduced features selected in the proposed feature selection approach.

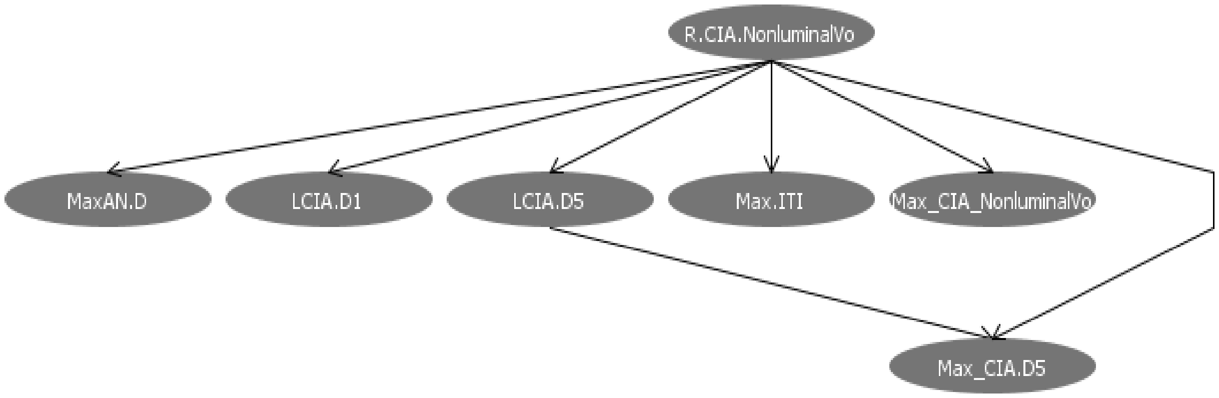


Figure S1. The high risk Bayesian network constructed using patients of the low risk group of the final seven reduced features selected in the proposed feature selection approach
