# Supplementary material for: Feature selection through validation and un-censoring of endovascular repair survival data for predicting the risk of re-intervention
Source: BMC Med Inform Decis Mak. 2017 Aug 3;17:115. doi: 10.1186/s12911-017-0508-3 (PMC5543447; doi:10.1186/s12911-017-0508-3)
Supplement: Supplementary file 2 — Comparative variable selection survival models and the generated Simulation Study. (PDF 180 kb) [file 12911_2017_508_MOESM2_ESM.pdf]

1. Comparative Feature model selection methods

1.1 Akaike information Criterion (AIC)

AIC was introduced by Akaike in 1977. It measures the quality of each candidate model. It is based on minimizing the Kullback Leibler distance which is the distance between the true and candidates' models. AIC takes into consideration the number of free parameters in the candidate's model and the goodness of their fit. The chosen model is the one that minimizes the AIC which is equivalent to the lowest distance to the true model [92] . It can be calculated using equation (1),

$$AIC = -2 \cdot \ln(L) + 2 \cdot K, \tag{1}$$

where, L is the maximum likelihood of the model given the data and K is the number of parameters in a given model.

1.2 Bayesian information criterion (BIC)

BIC was introduced by Schwarz in 1978. BIC also measures the quality of each candidate model. The penalty term in BIC is greater than AIC. BIC takes into account the number of observations available for each model which is not the case for AIC. Therefore, some researchers prefer it when they deal with models with small or different sample sizes. Again, the model which minimizes BIC is the one chosen [93] . It is calculated using equation (2),

$$BIC = -2 \cdot \ln(L) + 2 \cdot K \cdot \ln(n), \tag{2}$$

where; L is the maximum likelihood of the model given the data and K is the number of parameters in a given model, and n is the number of observations.

1.3 Least Absolute Shrinkage and Selection Operator (Lasso)

It was introduced by Robert Tibshirani in 1997 [94]. It is a L_1 penalized estimation method that shrinks the regression coefficients estimates β of Cox regression model towards zero using a tuning parameter λ which gives a penalty on their absolute values. This leads to removing the irrelevant variables from the predictive model. Shrinkage prevents over-fitting that may occur due to collinearity of the variables. The β coefficients of the predictive model are fitted by maximizing penalized partial log likelihood (*PPLL*) for all data with an absolute value LASSO penalty λ on β using equation (3)

$$PPLL_{\lambda}(\beta) = \sum_{i=1}^{n} \delta_i \left[(x_i^T \cdot \beta) - \log\left(\sum_{t_j \geq t_i} \exp(x_j^T \cdot \beta) \right) \right] - \lambda \|\beta\|_1, \qquad (3)$$

where, δ is the censor indicator for patient *i* with variables *x*. $\lambda \geq 0$ and $\|\cdot\|_1$ stands for L_1 norm. λ equal to zero means no shrinkage and infinity means infinity shrinkage. *Penalized* R- software package was used for implementing LASSO. The tuning parameter was selected using likelihood cross validation optimization method.

1.4 Smoothly Clipped Absolute Deviation (SCAD)

It was proposed by Fan and Li [46] as a concave penalty which corresponds to quadratic spline function with ties at λ and aλ. . SCAD is continuous and differentiable on (−∞, 0) U (0,∞) but singular at 0 with its derivatives zero outside the range [−aλ, aλ]. This function fulfills three properties when estimating regression coefficients β. These properties are the un-biasedness, sparsity and continuity. SCAD ends up with small coefficients being set to zero, while other coefficients are being penalized towards zero and finally holding the large coefficients as they are. SCAD can be solved using equation (4).

$$\hat{\beta}_j^{SCAD} = \begin{cases} \left|\hat{\beta}_j\right| - sign(\hat{\beta}_j) & if \left|\hat{\beta}_j\right| \langle 2\lambda \\ \{(a-1)\hat{\beta}_j - sign(\hat{\beta}_j)a\lambda/(a-2)\} & if\ 2\lambda \langle \left|\hat{\beta}_j\right| \leq a\lambda \\ \hat{\beta}_j & if \left|\hat{\beta}_j\right| \rangle a\lambda \end{cases} \quad (4)$$

2. Classification Models and Evaluation Metrics

The evaluation metrics that were employed to test the performance of the final selected model are discussed below.

- **Log Rank Test** is a popular statistical test used in clinical trials to distinguish between survival probabilities of two groups of patients that were either treated with different treatment, or have different risks of an occurrence of an event. This test uses chi squared test; which is the difference between patients that the event was observed and those that are expected over the square root of the variance of the expected ones of each group. The result of this test is a coefficient called p-value. A p-value smaller than 0.05 indicates that the two groups are significantly different, separable and discriminative [52].

- **Concordance Index** (CI) is the most frequently used predictive discrimination metric in the field of survival analysis for accessing model performance. It deals with the censoring nature that is found in survival data. CI [86] is the probability that, given two randomly selected patients, at least one of them must have experienced the event of interest at shorter follow up time; this patient must have higher probability of the event occurrence than the other. The greater CI indicates better performance and the prediction is more concordant and discriminative. A value of 1 signifies a perfect discrimination and concordance, while 0.5 shows no discrimination between the risk groups in predictions. In the recent decades, the CI

has become popular and used extensively especially in the field of biomedical research. For instance, searching the term "concordance index" in the PubMed database resulted in 4096 articles by the time this paper was written. Among these papers are [48,87-90] .

- **Uno's AUC estimator** is another metric used to compare the performance of survival models. The area under the ROC curves AUC is a well-known method to measure the performance of classifiers for standard data. Uno et al [91] has proposed Uno's AUC estimator metric which it similar to AUC, but used to evaluate survival models constructed with censored datasets. This metric depends on the inverse probability-of-censoring weights and is not limited to Cox proportional hazard model. It is used to deal with biasing issues that could occur due to censoring.

3. Simulation Study

A simulation study was performed beside the real EVAR dataset to demonstrate the effectiveness of the proposed feature selection algorithm. This study explains that a simplified model can be constructed with lower number of features using the proposed method. The simulated data consists of 27 variable and 1400 instances. The number of instance used for constructing the model and the feature selection process was 1000 and the remaining was used to test the performance of the simplified model after performing the proposed feature selection. The variables were created from the normal distribution [0,1]. The real predictive index of a linear risk score function f(x) is formed by the coefficients of [2; 2; 5; 3; 3; 8] for the first six variables while the remaining has no influence on risk-time event. Survival T and censoring C times were given to each instance depending on the predictor values. T is generated from

the exponential distribution exp(f(x)), and C is formed from the exponential distribution with parameter equals to 5. Afterwards, the survival data, $\{(t_i = \min(T_i, C_i), \delta_i = I(T_i \le C_i)) | i = 1, . . ., n\}$ with approximately 80% of right censoring.

4. Results of the Simulation Study

A Monte Carlo Simulation was performed and the average of the results of the proposed after 50 iterations has shown the effectiveness of the algorithm. This is because as shown in table 1, the number of feature has been reduced from 27 to 5.33 which are correctly recovered. However, the number of features that was falsely recovered is 0.67. Moreover, the final model has AUC of 0.653 which is the greater than that of the full model's AUC of 0.429. Moreover, the p-value is enhanced from 0.05 to 0.009 which indicates that the predictions using the reduced model can be separated significantly.

Table 1: Results of the proposed algorithm with the Simulation study data.

The proposed algorithm	Number of correctly recovered features	Number of falsely recovered features	p-value (Logrank)	Uno's AUC estimator
**All Features**	—	—	0.05 (0.04)	0.429 (0.014)
**Stepwise selection Features**	5.33	0.67	0.009 (0.0092)	0.653 (0.015)

The results of the Monte Carlo simulation of the proposed algorithm are also compared with that of the AIC, BIC, Lasso, and SCAD algorithms based on Cox's model. The results in table 2 show that the average number of features that were correctly recovered using the proposed algorithm (5.33) is better than AIC, BIC,

Lasso, and SCAD algorithms which are 5, 5.2, 5.36, and 5.2 respectively except for Lasso. Furthermore, the average number of features that were falsely recovered using the proposed algorithm (0.67) is lower than with AIC, BIC, Lasso, and SCAD algorithms are, 1.8, 2.4, 0.91, and 0.83 correspondingly. These numbers (the number of correctly and falsely recovered features) indicate that the proposed method outperforms popular survival variable selection methods. Moreover, the proposed algorithms has a better averaged AUC than other techniques 0.653 compared to 0.594, 0.592, 0.625, and 0.630 of the AIC, BIC, SCAD, and Lasso methods respectively. Finally, the averaged p-values of the log-rank test of the proposed algorithm

m are equal to 0.009, 0.044, 0.047, 0.058, and 0.02 of the AIC, BIC, SCAD, and Lasso methods respectively. These p values mean that the two risk groups are successively separated and distinguished except for the SCAD model.

Table 2: Results of the Simulation study data with the proposed algorithm compared with AIC, BIC, Lasso, and SCAD algorithms.

Algorithm	Number of correctly recovered features	Number of falsely recovered features	p-value (Log-rank)	Uno's AUC estimator
**Proposed Algorithms**	5.33	0.67	0.009 (0.0092)	0.653 (0.015)
**AIC**	5.0	1.8	0.044(0.07)	0.594 (0.057)
**BIC**	5.2	2.4	0.047(0.081)	0.592 (0.11)
**SCAD**	5.2	0.83	0.058(0.04)	0.625 (0.09)
**Lasso**	5.36	0.91	0.02(0.02)	0.630 (0.14)
